# Parent perceived barriers and facilitators of children’s adventurous play in Britain: a framework analysis

**DOI:** 10.1186/s12889-022-13019-w

**Published:** 2022-04-01

**Authors:** Brooke E. Oliver, Rachel J. Nesbit, Rachel McCloy, Kate Harvey, Helen F. Dodd

**Affiliations:** 1 School of Psychology and Clinical Language Sciences, University of Reading, Reading, UK; 2 Children and Young People’s Mental Health (ChYMe), College of Medicine and Health, University of Exeter, Exeter, UK

**Keywords:** Adventurous play, Children, Parents, Barriers, Facilitators

## Abstract

**Background:**

From a public health perspective there is growing interest in children’s play, including play involving risk and adventure, in relation to children’s physical and mental health. Regarding mental health, it is theorised that adventurous play, where children experience thrilling, exciting emotions, offers important learning opportunities that prepare children for dealing with uncertainty and help prevent anxiety. Despite these benefits, adventurous play has decreased substantially within a generation. Parents have a key role in facilitating or limiting children’s opportunities for adventurous play, but research identifying the barriers and facilitators parents perceive in relation to adventurous play is scarce. The present study therefore examined the barriers to and facilitators of adventurous play as perceived by parents of school-aged children in Britain.

**Methods:**

This study analysed data from a subsample of parents in Britain (*n* = 377) who participated in the nationally representative British Children’s Play Survey. Parents responded to two open-ended questions pertaining to the barriers to and facilitators of children’s adventurous play. Responses were analysed using a qualitative Framework Analysis, an approach suitable for managing large datasets with specific research questions.

**Results:**

Four framework categories were identified: Social Environment; Physical Environment; Risk of Injury; Child Factors. Social Environment included barriers and facilitators related to parents, family and peers, as well as community and society. Dominant themes within the Social Environment related to perceptions about the certainty of child safety, such as supervision and the safety of society. Beliefs about the benefits of adventurous play for development and well-being were also important in the Social Environment. Physical Environment factors focused on safety and practical issues. Risk of Injury captured concerns about children being injured during play. Child Factors included child attributes, such as play preference, developmental ability and trait-like characteristics.

**Conclusions:**

Improved understanding of what influences parent perceptions of adventurous play can inform public health interventions designed to improve children’s opportunities for and engagement in adventurous play, with a view to promote children’s physical and mental health.

**Supplementary Information:**

The online version contains supplementary material available at 10.1186/s12889-022-13019-w.

## Background

Children’s play, including play involving risk challenge and adventure, is increasingly recognised from a public health perspective as important for children’s physical and mental health [1]. Adventurous play, synonymous with risky play, is defined as “child-led play where children experience subjective feelings of excitement, thrill and fear; often this occurs in the context of age-appropriate risk-taking” ([2] p.1). It includes activities such as climbing, jumping or running at great speed [3] and tends to occur more during outdoor than indoor play [2, 4].

Research has shown that adventurous, outdoor play has positive effects on children’s physical and mental health [5]. For example, a systematic review exploring risky play in children aged 3–13 years concluded there were several benefits for children’s health, including increased physical activity, reduced sedentary behaviours, increased social competence and improved creativity and resilience [5]. Adventurous play has also been theorised as protective against the development of fears [6] and anxiety [2], by providing natural exposures and corresponding learning opportunities where children can learn about uncertainty, coping and arousal. Importantly, research shows that children enjoy the emotions that they experience during adventurous play. Common feelings elicited are a sense of thrill and exhilaration which borders on fear, and feelings of pride and achievement after the play [4, 7, 8].

Research examining the benefits of adventurous play for children’s development indicates that this type of play may support children’s mental well-being and development [9]. For example, in Lavrysen and colleagues, [10], two school classes of children aged 4–6 years were given opportunities to engage in risky play over three months, including play at great height and speed. Post-intervention, significant improvements were observed in teacher ratings of children’s self-esteem, conflict sensitivity and concentration.

Despite the recognised benefits of adventurous play and the knowledge that children enjoy playing in this way, there is convincing evidence that engagement in adventurous play across westernised countries has declined significantly in recent decades [9, 11]. For example, in a survey of mothers in the USA, 60% reported that they played in adventurous ways as children, but only 22% of their children played in this way [12]. Large surveys conducted in Australia [13] and New Zealand [14] provide further support for this decline in adventurous play. For example, Jelleyman and colleagues [14] found that less than half of the parents surveyed reported that their children engage in risky play activities on a regular basis. Given the evidence that adventurous play has benefits for health, the declines seen may therefore have considerable implications for children’s physical and mental health [2, 5].

When considering children’s declining opportunities for adventurous play, a range of influences are likely to be important, as highlighted by socio-ecological and psychological models [15, 16]. Parents in particular have a core role in providing or restricting children’s opportunities for adventurous play [17, 18]. The role of parents is highlighted by the British Children’s Play Survey [19], where parents’ tolerance of risk and attitudes to child risk-taking were positively associated with children’s time spent playing adventurously. There has also been some emerging evidence for the role of parenting styles, in particular overprotective styles, in predicting whether children take risks in their play or not [20]. Parent sex may also be important, with fathers, on average, more comfortable with child risk-taking than mothers [21] (see also [22]).

Previous research indicates that parents often hold positive beliefs about the benefits of adventurous play. For example, Jelleyman and colleagues [14] reported that the majority of parents believed risk in play supports the development of risk management skills and development more generally. This aligns with the findings of Little and colleagues [23] and Little [24], where parents often stated that risky play fosters learning and development and supports children’s self-esteem. This highlights a disconnect between parent positive beliefs and children’s play opportunities. It is unclear therefore how interventions should address the decline in children’s adventurous play; interventions are unlikely to be successful if they focus solely on the benefits of adventurous play for children given that parents likely already hold these positive beliefs. Instead, interventions will need to address the barriers that prevent parents from translating positive attitudes towards adventurous play to increased opportunities for their children.

Research regarding what parents perceive to be the barriers and facilitators of children’s adventurous play is scarce. Some inferences can be drawn from small scale studies exploring parent perceived barriers to risky play in young children [25, 26] and large surveys that have explored unsupervised outdoor play in nature [11], as well as children’s independent mobility, defined as the freedom children have to travel and play around their local neighbourhood [27, 28]. Though outdoor nature play and independent mobility are not synonymous with adventurous play, both afford increased opportunities for it [2]. Frequently identified barriers across these studies include concerns about road safety [25] and “stranger danger” [11, 29, 30]. Concern about the risk of physical injury involved in adventurous play has also been identified as a key barrier for parents [25, 26, 31].

Other previously cited barriers in relation to children’s safety when playing are the absence of adult supervision [32, 33], the potential for bullying [27] and perceptions of low social cohesion in neighbourhoods, leading to an expectation that neighbours will not look out for each other’s children [17]. Practical barriers have also been acknowledged, such as having the time to facilitate adventurous play [25], poor weather conditions [14] and poor accessibility to play spaces or activities [32, 34].

Some parents have also identified barriers related to themselves, such as concerns about other parents’ judgements of them [35] and their own anxieties [24]. Children’s attributes and preferences have been highlighted in some studies, with some parents citing difficulties encouraging their children to play outdoors, unsupervised or adventurously because they do not enjoy it, or because they prefer to play indoors using technology [12, 33]. Together, the barriers ascertained by parents across several countries demonstrates the multifaceted nature of parents’ perceptions in relation to their children’s play. This highlights the challenge of supporting parents to overcome these barriers and the difficulty in translating this into increased play opportunities.

In previous research, findings related to barriers and facilitators have typically been obtained using multiple choice questions where a set of predefined options are provided or via agreement ratings against predetermined statements. An example is the New Zealand State of Play Survey, where parents rated their level of agreement with statements about the barriers of risky play from *‘strongly disagree’* to *‘strongly agree’*, such as *‘unsupervised activities increase the chance of injury’* [14]. Whilst this provides an efficient way of collecting quantitative data, it has limited utility when inferring what is most pertinent or important to parents and necessarily restricts responses to options the researchers have considered. A further limitation is that evidence has been gathered almost exclusively on barriers, rather than also gathering information on the perceived facilitators. This information is required to improve understanding of what might support parents to encourage their child to play in an adventurous way. Asking parents to respond to open-ended questions regarding both barriers and facilitators of children’s adventurous play would have greater utility for the design of future public health interventions.

The aim of the present study was to explore and identify the barriers and facilitators for parents in Britain with regards to encouraging their children to play adventurously. Data was collected as part of the British Children’s Play Survey (BCPS) [19], a nationally representative survey of parents and caregivers with primary school-aged children. Two open-ended questions asked parents to identify the barriers and facilitators they perceive when encouraging their children to engage in adventurous play. Responses were analysed using Framework Analysis [36].

## Method

This paper analysed data collected as part of the BCPS. For full details of the survey and the recruitment of participants, see Dodd and colleagues [19].

### Design

This paper describes the analysis of a subset of written responses to two open-ended questions included in the BCPS. To explore parents’ responses to the open-ended questions asking about barriers to and facilitators of adventurous play, a qualitative approach to sampling and data analysis was adopted.

### Participants and procedure

The BCPS centred around play in primary school-aged children. Participants were a nationally representative sample of 1919 parents and caregivers of children aged 5–11 years old, living in Britain. Participants were recruited via YouGov, a UK public opinion research company. Participants completed the survey online and anonymously, ensuring confidentiality. Any identifying information, such as individual names or names of places, included in participants’ responses were removed before analysis to further ensure confidentiality. The methods and procedure were approved by the University of Reading School of Psychology  and Clinical Language Sciences Ethics Committee (2020-003-HD). This study analysed data from a subsample of 377 parents and caregivers. 22 parents and caregivers did not provide any answers to the questions analysed and 9 expressed that they were unsure how to answer. For simplicity, we refer to participants as parents.

### Sampling

It was not feasible to analyse in depth all 1919 responses to the open-ended questions included in the BCPS, and so, consistent with our methodological design, a sub-sample was selected using purposive sampling [37]. The aim was to achieve a sample of parents who were diverse on key demographic characteristics that were relevant: parent sex, child sex, child age group (younger: 5-7-years-old; older: 8-11-years-old), parent education and parent ethnicity. The final sample included 377 parents. Data saturation was reached and so no further responses were included in the sample. The demographic characteristics of the final sample can be seen in Table 1. For further details on how these parents were selected see supplementary material Additional file 1.

**Table 1:** Table 1

Demographic characteristics of the final sample	
**Characteristic**	***N*** **(%)**
Parent sex	377
Male	185 (49%)
Female	192 (51%)
Child sex	377
Male	185 (49%)
Female	192 (51%)
Child age group^a^	377
Younger	163 (43%)
Older	214 (57%)
Parent education level	377
Lower	101 (27%)
Medium	119 (32%)
Higher	157 (42%)
Parent ethnicity	377
White British	103 (27%)
Other White ethnicity	56 (15%)
Mixed ethnicities	37 (10%)
Asian/Asian British	51 (14%)
Black/Black British	21 (6%)
Other ethnicities	12 (3%)
No ethnicity information given	97 (26%)

^a^ Younger children were aged 5-7-years-old and Older children were aged 8-11-years-old

### Measures

As part of the survey, parents were given a definition of adventurous play and were asked two open-ended questions, which were designed by the authors of the BCPS [19]. The first question asked, ‘Which factors, if any, allow you to let / encourage your child aged 5-11 to play in an adventurous way?’. The second question asked, ‘Which factors, if any, make it difficult for you to let / encourage your child aged 5-11 to play in an adventurous way?’. Parents were encouraged to provide as much detail as possible in their answers. Answers aimed to elicit responses to the barriers to and facilitators of adventurous play. The data is available here: 10.5255/UKDA-SN-8793-1. The wider survey included detailed demographic questions.

### Data analysis

Responses to the two open-ended questions were analysed using Framework Analysis, a qualitative analysis technique that involves a systematic yet flexible process of organising, charting and arranging data in line with key themes and issues [36]. Framework Analysis includes five stages, which are discrete but highly connected. The process involves familiarisation with the dataset, identification of a thematic framework, indexing or applying the thematic framework to the data, charting the indexed data into charts of themes and mapping and interpretation of the dataset as whole [36]. This method was chosen due to its suitability for analysing large samples and its position as an approach which is not bound by a particular philosophical perspective [36]. It is also suited to research with specific research questions and a priori ideas, while equally encouraging an inductive, data-driven approach [38].

The five stages of Framework Analysis were flexibly applied, to ensure the analysis was data-driven. NVivo 12 (QSR) and pen-to-paper methods facilitated the analysis. The analysis was led by the lead author BO, who began with familiarisation of the data and then coded the data using the research aim as a guide. Reflective notes were taken during the analysis to stimulate reflection at each stage, as recommended by Ritchie and Spencer [36]. An initial overarching framework of ‘barriers’ and ‘facilitators’ was applied during the initial stages of analysis but was later withdrawn. We chose to withdraw this framework on reflection that being able to identify the overall factors that affect parent decisions may have greater utility in terms of interpretation and recommendations for policy and intervention. A preliminary thematic framework was then identified using an inductive approach and thematic coding. The framework went through several iterations, following discussions and reflections with co-authors. The framework was derived inductively, with the aim of building a novel framework and to ensure a true reflection of parents’ responses.

**Fig. 1:**
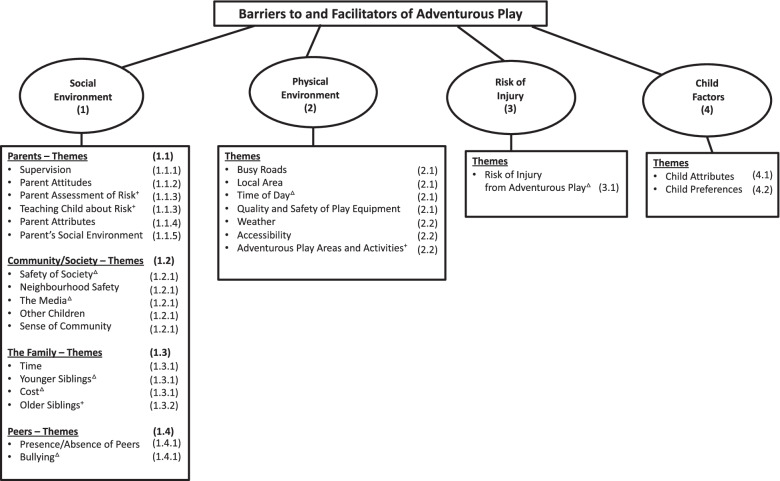
A framework capturing parent perceived barriers to and facilitators of adventurous play. Note. ^+^Themes identified as facilitators only. ^△^Themes identified as barriers only. Numbers in brackets highlight the results section in which the framework categories and themes are discussed

 Codes were synthesised and then grouped according to dominant themes. The process was iterative and utilised notes taken throughout the analysis to stimulate reflection. Codes and themes were presented and discussed regularly with co-authors, contributing to refinement of the framework and supporting the credibility of the analysis [39, 40]. An example is reflected in the ‘Risk of Injury’ category (Fig. 1). Originally, this category was included as a theme within the ‘Physical Environment’. On reflection and discussions, it was agreed that parents were referring to the adventurous play activity itself, as opposed to the risk of injury posed from the physical environment. It appeared dominant in many parents’ responses and therefore felt important as a distinct framework category.

 During the first stages of analysis, it was noted that parents often recorded “safety” or “safe environment” in response to both questions. Whilst this highlighted the dominance of safety considerations underlying parents’ thoughts about adventurous play, it was difficult to determine whether such answers were specific to the type of play or play in relation to the physical or social environment. Due to this difficulty, and to ensure responses were not forced into the framework, responses here were coded into a separate, safety theme. This theme was not included in the final framework but greatly supported the final stages of analysis when interpreting the data by emphasising the dominance of underlying safety considerations.

Charting of the framework was completed but proved challenging due to the number of parents sampled. To support the deeper exploration of differences in responses amongst parents with different socio-demographic characteristics, matrix coding queries were executed. These differences are highlighted throughout the Results section. This supported the final stages of analysis, where the research team explored the meaning underlying parents’ responses in more depth.

It is acknowledged that the research team’s prior knowledge, assumptions and experiences inevitably affected the analysis and so, reflexivity was essential to ensure the research was rigorous and credible [41, 42]. The research team all had knowledge of adventurous play from a psychological perspective and were working with the same definition of adventurous play [2]. It is therefore likely that the research team were inclined to view adventurous play overall as a positive phenomenon with which children should have opportunities to engage. Also, some members of the research team were parents, others were not parents and all members had a range of personal experiences of adventurous play. Collectively, these assumptions and experiences may have influenced the nature of the analysis.

## Results

During analysis, it became apparent that adventurous play may have different meanings for parents. For some, it appeared primarily related to the perception of risk in the play. For others, it appeared synonymous with play in natural spaces (e.g. forests, woodlands, near water) or autonomous play, including independent mobility. The remainder appeared to perceive adventurous play as a combination of some or all of these elements. Parents’ views were captured in four framework categories (see Fig. 1); Social Environment, Physical Environment, Risk of Injury and Child Factors.

### 1. Social environment

The Social Environment category involved the most themes. The Social Environment included factors pertaining to Parents, Community and Society, Family and Peers. Themes within each are discussed below.

#### 1.1 Parents

Parent factors were one of the most commonly referenced factors within the Social Environment, particularly in terms of facilitators. Themes identified within parent factors were: Supervision, Parent Attitudes, Parent Assessment of Risk, Teaching the Child about Risk, Parent Attributes and Parent’s Social Environment (Fig. 1). Factors which increased or decreased perceptions about the certainty of child safety coupled with beliefs about the benefits of adventurous play appeared to underlie this theme. These insights were captured predominantly in the themes Supervision and Parent Attitudes.

##### 1.1.1 Supervision

Supervision was regularly cited as both a barrier and facilitator, with lack of adult supervision forming a key barrier for parents with regards to adventurous play. Parents’ views on inadequate supervision ranged from lack of direct supervision, to not knowing where their child is playing. Similarly, as a facilitator, presence of supervision was commonly identified but varied in terms of the degree of supervision, from direct adult supervision, to playing in close proximity to the home, to being able to contact their child via a mobile phone. Adult supervision as a facilitator for adventurous play tended to be more of a consideration for parents with younger children in this sample.

*“You can’t just let them go and play. You can’t let them out of your sight.”* (P154; Mother of younger boy, higher education level, White British).

*“Adult supervision not to dictate and control the play but to be watching in case they’re needed.”* (P94; Father of younger girl, medium education level, White British).

*“Need to know where he is, who with, what time he’ll be back, ideally with his mobile phone.”* (P21, Father of older boy, lower education level, White British).

##### 1.1.2 Parent attitudes

Beliefs about the benefits of adventurous play for children’s learning and development, health and well-being were often referred to as facilitators. These factors were grouped under the theme of Parent Attitudes. Most parents with positive attitudes acknowledged the benefits of adventurous play for learning and development, including the ability to assess risk and problem solve. Also present but appearing to be less readily acknowledged by parents was the benefits for physical and psychological health and well-being, such as confidence and resilience. Some parents attributed their attitudes to their occupation or their own play experiences as a child. These attitudes aligned with some who identified their self-attributes as parents who were focused more on following the child’s interests and who did not want to be overprotective. Only one parent appeared to hold a negative attitude about adventurous play, suggesting it is a “bad idea” that represents “irresponsible parenting”.

*“Build confidence, learn problem solving, being active.”* (P141; Father of older girl, medium education level, Asian ethnicity).

*“I feel that without adventurous play he will be less likely to develop the ability to assess risk. His ability to function independently as he grows will be inhibited if he’s not allowed to make good calculated decisions.”* (P338; Mother of younger boy, higher education level, no ethnicity information).

*“Knowing that they need some form of adventurous play as it helps them learn the dangers of different activities, burning off energy.”* (P72; Mother of younger boy, medium education, White British).

*“Want to protect my child. The idea of “adventurous play” is false and reckless. Irresponsible. It’s a bad idea. It’s irresponsible parenting. Seriously.”* (P247; Father of older boy, higher education level, Asian ethnicity).

##### 1.1.3 Parent assessment of risk; teaching the child about risk

Whilst two separate themes were identified, these were less frequently identified than the themes aforementioned and are therefore presented together for ease. The two themes were parents’ own assessment of the level of risk in the adventurous play activity and teaching children about assessing and managing risk. These were identified as facilitators only.

*“All activities whatever time of year and in whatever location are supervised and assessed for risks and competence by both parents and we all do it together.”* (P320; Mother of older girl, medium education level, no ethnicity information).

*“Safety of environment (risk assess the area to mitigate risk).”* (P231; Father of older girl, higher education level, Mixed ethnicity).

*“I educate my child on the dangers of certain activities, to make him aware of the possible dangers.”* (P69; Mother of younger boy, medium education level, White British).

##### 1.1.4 Parent attributes

A barrier occasionally identified was parent attributes. These included self-attributes as a “worrier” or “anxious”, or attributes of their child’s other parent. Only male parents cited their child’s other parent when describing attributes as a barrier. Acknowledgement of parenting styles were also described within this theme, with responses pertaining to overprotective or controlling parenting, which bears similarity to and appears coherent with the attributes cited above.

*“I’m a very anxious person so tend to worry a lot about their safety.”* (P119; Mother of older boy, medium education level, Mixed ethnicity).

*“My wife’s anxiety of risk.”* (P203; Father of older boy, higher education level, Other White ethnicity).

*“I don’t encourage adventurous play I am over protective.”* (P16; Mother of older girl, lower education level, White British).

##### 1.1.5 Parent’s social environment

Present for a small minority of parents, and primarily a barrier, were perceptions of their social environment. Specifically, some parents identified the judgements others may make of them as parents as a barrier. One parent explicitly recognised that if they perceived less judgement from others, this would help them encourage more adventurous play. One parent appeared to seek support from their social environment, commenting that the presence of parents who were less risk averse than them helps them encourage their child to play adventurously.

*“Concerns from other parents.”* (P172; Father of younger boy, higher education level, White British).

*“Judged if letting them take risks by other less risk taking parents.”* (P346; Mother of younger girl, higher education level, no ethnicity information).

*“Others there who are less risk averse than me.”* (P157; Mother of younger boy, higher education level, White British).

#### 1.2 Community and society

Perceptions of the Community and Society was a further identified factor within the Social Environment. Five themes were identified.

##### 1.2.1 Safety of society; neighbourhood safety; the media; other children; and sense of community

Key themes within the Community and Society were: Safety of Society, Neighbourhood Safety, The Media, Other Children and Sense of Community (Fig. 1). Given that they all relate to children’s safety, these five themes are described together but they are considered separate themes within the framework.

Overall, more barriers than facilitators were suggested across these five themes. A considerable number of parents cited the safety of society as a barrier. These concerns tended to be more of a consideration for parents with older children. The Safety of Society theme included concerns about “stranger danger”, antisocial behaviour or crime and perceptions of general neighbourhood safety. This aligned with parents who cited an absence of community as a barrier, believing that neighbours do not look out for each others’ children like they used to. Linked to these themes was the role of the media. Some parents suggested the reporting of accidents and incidents in the news makes it difficult for them to encourage their children to play adventurously. A small number of parents also considered other children’s involvement in the play in their responses, with one parent citing concerns about “young other children” being present and two parents citing concerns about the presence of “older kids”.

*“I am worried that he may get lost or be taken.”* (P36; Mother of older boy, lower education level, Other White ethnicity).

*“A lot of bad people around.”* (P143; Mother of older girl, medium education level, Black ethnicity).

*“Horrible stories you hear when kids have been allowed to play without adult supervision.”* (P319; Mother of older girl, medium education level, no ethnicity information).

When the Community and Society were cited as facilitators, it tended to be by parents who perceived a strong sense of community, a generic perception of living in a safe neighbourhood and a sense of certainty that neighbours will look out for each others’ children. Collectively, the themes within the Community and Society appeared to operate to increase or decrease perceptions of child safety during adventurous play.

*“We also live on a quiet cul de sac with a number of young families. This unique arrangement allows us to let our children explore the local environment with minimal supervision.”* (P204; Father of older boy, higher education level, Other White ethnicity).

*“Safe neighbourhood with good neighbours that communicate about kids and where they are.”* (P341; Mother of older boy, higher education level, no ethnicity information).

#### 1.3 The family

Family life considerations were another factor of the Social Environment, but were cited less often than Parent and Community factors. When the family was identified, this was generally to acknowledge the barriers. Key themes within The Family were: Time, Younger Siblings, Cost and Older Siblings (Fig. 1).

##### 1.3.1 Time; younger siblings; cost

Whilst these themes are separate and distinct from one another, most responses within these themes related to practical considerations about adventurous play, and so are presented together. Time was identified as both a barrier and facilitator. Having a lack of time was cited as a barrier and in contrast, having more time was cited as a facilitator. Having another, younger child was identified only as a barrier in this sample, due to the practicalities of considering activities that are appropriate for all siblings or due to safety concerns that they may want to try the same activity as their older sibling. Although not present in the majority of parents’ responses, the cost of adventure play spaces and having the energy to facilitate adventurous play were barriers for some.

*“Time constraints as parents due to full time work commitments / home related commitments can make it difficult to allow our children daily adventurous play.”* (P332; Father of younger girl, medium education level, no ethnicity data).

*“Have to factor in things that are suitable for his younger siblings too.”* (P71; Mother of younger boy, medium education level, White British).

*“Cost of some adventure places.”* (P73; Mother of older boy, medium education level, White British).

##### 1.3.2 Older siblings

In this sample, older siblings were identified as facilitators of adventurous play only. Here, the presence of older siblings for the child to play with was identified as a facilitator; underlying this theme may be increased perceptions of child safety during adventurous play.

*“He is allowed with his elder brother.”* (P218; Mother of older boy, higher education level, Mixed ethnicity).

*“Playing with other children especially older siblings.”* (P334; Father of older girl, medium education level, no ethnicity information).

#### 1.4 Peers

Peers were more readily cited as facilitators than barriers for adventurous play. Key themes were: Presence or Absence of Peers and Bullying (Fig. 1). As these themes were less frequently identified than other themes within the framework, they are presented together for ease.

##### 1.4.1 Presence/absence of peers; bullying

The presence of known, trusted peers was considered a facilitator of adventurous play. Safety considerations were thought to underlie these responses, possibly by increasing certainty about safety or certainty that someone can help in case of an emergency. Unknown or untrusted peers, and the possibility of bullying were sometimes listed as barriers, as well as the practical considerations of not having or knowing any peers to play with, though this did not emerge to be a general consensus across parents.

*“When she’s with friends.”* (P32; Father of older girl, lower education level, White British).

*“With other trusted children.”* (P103; Mother of older boy, medium education level, Other White ethnicity).

*“Not having/knowing of any children locally for him to play with.”* (P214; Mother of younger boy, higher education level, Mixed ethnicity).

### 2. Physical environment

Although less commonly identified than the Social Environment, the Physical Environment also represented a consideration for many parents. Barriers in the Physical Environment were more regularly cited than facilitators, suggesting parents had more ideas about how the perceived physical environment hinders their encouragement of adventurous play. Key themes were: Busy Roads, Local Area, Adventurous Play Areas, Time of Day, Quality and Safety of Play Equipment, Weather and Accessibility (Fig. 1).

#### 2.1 Busy roads; local area; time of day; quality and safety of play equipment

These themes within the Physical Environment, whilst they are stand-alone themes, all related to safety and so will be presented together. In particular, busy roads, the perceived safety of the local area lived in and the play setting were most commonly identified as barriers. Concerns about the busyness of roads tended to be more of a consideration for parents with older children. Though uncommon, some parents also considered the time of day, particularly dark nights as a barrier.

*“I think the biggest challenge for any parent or child is road traffic.”* (P272; Father of older girl, higher education level, Other ethnic group).

*“Location of our home, dangerous trunk road outside house, very rural setting.”*^*1*^ (P156; Mother of younger boy, higher education level, White British).

^**1**^ A trunk road is a major road built for travelling long distances, for example a dual carriageway.

*“Dark nights.”* (P296; Father of older boy, lower education level, no ethnicity information).

Aspects of the Physical Environment that increased perceptions of child safety were also facilitators for parents, but were less often explicitly stated. Safe play equipment, well-regulated and monitored play areas, perceptions of living in a safe home area with little traffic and safe access to adventurous play opportunities were considered as facilitators.

*“My neighbourhood is rural and there’s no busy roads, lots of green spaces so I feel safe letting him go out to play in this area.”* (P35; Mother of older boy, lower education level, Other White ethnicity).

*“We are lucky to live in a very wild place where she can play safely unsupervised away from road traffic.”* (P272, Father of older girl, higher education level, Other ethnic group).

#### 2.2 Weather; accessibility; adventurous play areas and activities

The themes included here are also separate, distinct themes but all related to practical considerations about adventurous play, and so are presented together. These themes were identified as both barriers and facilitators. As expected, poor weather conditions and poor access to adventurous play facilities characterised barriers, whereas good weather conditions and easy access to places that afford adventurous play characterised facilitators. The places and activities that parents perceived to facilitate adventurous play varied considerably, with some parents citing “forests” or “beaches” pertaining to natural spaces, others citing “trampolines”, “climbing frame in garden” and some citing organised team sports, “game of cricket”.

*“Weather often makes it difficult to have a good time playing outside in this country.”* (P108; Mother of younger girl, medium education level, Any other White background).

*“There is little adventurous toys or places to be this.”* (P79; Mother of younger girl, medium education level, White British).

*“There’s not many adventurous play things nearby without driving.”* (P35; Mother of older boy, lower education level, Other White ethnicity).

### 3. Risk of injury

#### 3.1 Risk of injury from adventurous play

Central to many parent’s responses about the barriers of adventurous play were concerns about the risk of injury that accompanies it (Fig. 1). This framework category captured both parent’s perceptions of how likely it is that the child will hurt themselves during the play and also worries and fears about the child getting hurt from the play.

*“Worrying that the risks they take may result in injury.”* (P72; Mother of younger boy, medium education level, White British).

*“Risk of injury/harm.”* (P234; Mother of older boy, higher education level, Asian ethnicity).

*“Fear of them getting seriously hurt.”* (P125; Father of older girl, medium education level, Mixed ethnicity).

### 4. Child factors

Perceptions of the child also featured in parents’ responses. Key themes were: Child Attributes and Child Preferences (Fig. 1).

#### Child attributes

Child Attributes captured a variety of barriers and facilitators related to the child. This included considerations related to typical and atypical developmental and physical abilities, age and sex, as well as perceptions of trait-like characteristics. For parents who viewed child attributes as a barrier, the considerations that were most present were those related to developmental and physical abilities. Although we didn’t explicitly sample for parents who had children with additional needs, some parents identified barriers relating to their child’s additional needs, citing that because of this, adventurous play was not possible or was more difficult to facilitate.

For parents of typically developing children, barriers included both perceptions of capabilities relating to the adventurous play itself, such as not yet being able to assess and manage risks, or perceptions of the child as “clumsy” or “accident prone”. Others focused on trait-like characteristics, such as shyness or lack of confidence. Some barriers were noted in relation to playing independently, such as being too trusting of strangers.

 Age of the child was considered a barrier by some parents, but the nature of this varied. Some parents reported their child is too young to play adventurously and others reported concerns in relation to playing unsupervised. Child sex was reported by one parent as a barrier, with a father reporting that he finds it harder to let his daughter play unsupervised. No other parents explicitly identified child sex as a barrier or facilitator.

*“Severe disability means this isn’t possible.”* (P373; Foster father of younger boy, higher education level, White British).

*“She is scared to do this.”* (P110; Mother of older girl, medium education level, Other White ethnicity).

*“I think he is young for adventures.”* (P321; Father of younger boy, medium education level, no ethnicity information).

When regarded as a facilitator, perceptions of child attributes almost exclusively focused on views of the child as needing or having a sense of responsibility and maturity to manage risks and keep themselves safe. Some parents considered the developmental capabilities of the child as a facilitator, such as assessing how ready the child is to play adventurously or ensuring the child plays appropriately for their age and developmental level.

*“He is a sensible child and knows boundaries.”* (P311; Mother of older boy, medium education level, no ethnicity information).

*“She is sensible and conscious of risk.”* (P179; Father of younger girl, higher education level, White British).

*“How ready she is to play in an adventurous way.”* (P124; Father of younger girl, medium education level, Mixed ethnicity).

#### 4.2 Child preferences

Views of the child’s play preferences were also identified by some parents as barriers and facilitators. With regards to barriers, responses tended to focus on perceptions that their child does not enjoy adventurous play. Regarding facilitators, responses were focused on perceptions that their child enjoys playing adventurously, with some parents specifying the types of activity their child enjoys, such as climbing or swimming.

*“My child doesn’t really enjoy adventurous play even when given the opportunity.”* (P71; Mother of younger boy, medium education level, White British).

*“My kids love it.”* (P10; Mother of a younger girl, lower education level, White British).

*“She loves climbing, walls, trees the big climbing frame at the park. Running and jumping off things, trying to cartwheel at the beach.”* (P78; Mother of younger girl, medium education level, White British).

## Discussion

This is the first study to explore the barriers to and facilitators of adventurous play for parents of school-aged children in Britain. The findings highlight that parents perceive a multitude of barriers and facilitators. Key barriers included concerns about the safety of society, concerns about the risk of injury from play and concerns regarding child attributes, including developmental capabilities and traits. Key facilitators included positive attitudes and beliefs about the benefits of adventurous play. This discussion focuses on the factors that appeared to stand out as being most important to parents and those that have particular relevance for policy and public health intervention design.

A common facilitator of children’s adventurous play was parents’ positive beliefs and attitudes towards its benefits, which they identify as offering children learning and development opportunities. Specifically, many parents cited improved risk assessment and problem-solving skills as positive outcomes. It was uncommon for parents to state that adventurous play was not worthwhile. Although less readily identified, parents also considered adventurous play as beneficial for children’s well-being, including their confidence and resilience. These findings are in keeping with several studies which report that parents hold positive attitudes towards adventurous play [14, 24].

These positive attitudes towards adventurous play compete with a range of perceived barriers. The most cited barriers related to concerns for child safety, specifically, the absence of supervision, concerns regarding the safety of society, including “stranger danger” and concerns regarding the risk of injury. Some consistencies are seen with studies such as McFarland and Laird [25, 31], where parents identified concerns about the risk of injury as a barrier to risky play and concerns about “stranger danger” as a barrier to unsupervised, risky play. Interestingly, in the definition of adventurous play given to parents, we did not state that it was necessarily unsupervised, but the responses indicate that some parents interpreted adventurous play to involve play without supervision.

Barriers related to the physical environment, such as busy roads, accessibility of play areas and the weather were cited but appeared less central than in previous research (e.g. Jelleyman and colleagues [14]). This may be because, in previous research, findings relating to barriers have often been obtained by asking parents to select from a set of predefined options or via agreement ratings against predetermined statements. The current study differed in that parents were invited to respond to open-ended questions and it may be that the weather and roads, while important, are less salient to parents than barriers they chose to describe. A further explanation could be that previous studies often asked parents about outdoor play specifically, or focused on examples of risky play that occur outdoors, such as climbing or playing near water [14, 30], priming parents to think about the conditions required for outdoor play. By contrast, in this study parents were given a definition of adventurous play that did not specify its occurrence exclusively in indoor or outdoor settings.

In general, it appeared that parents held similar views on adventurous play despite different socio-demographic characteristics. Some barriers however, such as concerns about busy roads and societal safety appeared to be more frequently perceived by parents of older children. This could infer that, although independent play or mobility and adventurous play are not considered theoretically synonymous, parents may perceive them as synonymous with increasing child age.

 Some of the parents reported that their child’s additional needs were barriers to adventurous play. This aligns with findings from the BCPS that children with additional needs spent significantly less time playing adventurously than their typically developing peers [19]. Additionally, Beetham and colleagues [43] found parents of children with additional needs had a lower tolerance of risk in relation to their children’s play than parents of typically developing children. When asked to select activities that caused them discomfort, these parents selected activities that involved a lower risk of injury than those selected by parents of typically developing children, such as swimming underwater compared to climbing trees. Parents in the current study sometimes referred to their child’s specific diagnosis in their responses, such as autism or learning difficulties, whereas others wrote about their child’s ‘disability’ more broadly. It seems likely that the barriers and facilitators will differ depending on the nature of the child’s additional needs, however the details of these differences and why could not be elucidated in parents’ responses in this study. Equal access to play opportunities for children with disabilities is an under-researched area and so, future research could work more closely with parents and children to inform how we can better provide for their needs.

### Embedding the findings in psychological theory and evidence

Although the current study approached data analysis in an inductive way, the findings can be situated and contextualised within psychological theory and evidence related to child development. Particularly relevant for contextualising our findings is Bronfenbrenner’s ecological systems theory of development [15]. Bronfenbrenner’s model considers development as being influenced by interactions operating between the child and their characteristics, influences in the child’s immediate environment such as parents and peers, as well as those in the wider social and cultural environment. This model has also been applied to help understand children’s play opportunities [31].

#### The child

Child individual differences, such as the temperamental trait behavioural inhibition and individual differences in sensation seeking, are relevant to the current findings. Behavioural inhibition is a temperament trait, first defined by Kagan and colleagues [44], as the tendency to be cautious, withdrawn and shy in unfamiliar situations. Given these characteristics, it is likely that children with this trait will be less inclined to play adventurously than their uninhibited counterparts. These findings align with the current findings, with child individual differences featuring in many parents’ responses. When identified as a barrier, parents cited traits related to shyness as well as the child’s dislike for adventurous play, which bears similarities to the characteristics of behavioural inhibition.

Individual differences in the propensity to seek out novel, complex and intense experiences, known as sensation seeking [45], have also been found in relation to children’s inclination to take risks. Children who are high sensation seekers have been shown to take significantly more risks in their play than low sensation seekers [46], which means these children may be more inclined to seek adventurous play opportunities. The current study found that when parents identified child individual differences as facilitators, they often focused on the child’s enjoyment of adventurous play or their trait as a natural risk-taker. These resemble the characteristics of sensation seeking and suggests that these children may therefore need less encouragement to engage in adventurous play.

#### Parents

Parent factors are recognised as a central influence within the child’s immediate environment [15]. Research into individual differences may have relevance to the current findings, particularly differences in Intolerance of Uncertainty, which refers to trait-like differences in responses to uncertainty [47] and research showing that overprotective or overinvolved parenting styles are associated with parent anxiety [48]. It seems likely that parents who are high in Intolerance of Uncertainty and who have an overinvolved parenting style may place restrictions on their children’s activities, including their play. This would limit children’s opportunities for exposure to uncertainty and the opportunity to develop skills on how to cope with and manage uncertainty [49–51]. Parents who have difficulty coping with uncertainty or managing their own anxiety may therefore have more barriers to overcome in relation to their children’s adventurous play than those who have less difficulty coping with uncertainty.

 These differences align with the current findings, where it appeared that an overriding consideration for many parents was the importance of feeling certainty about their child’s safety. Many of the barriers identified inferred feelings of uncertainty, with concerns about child safety exacerbated by factors that increased uncertainty, such as the absence of supervision. For parents who described themselves as anxious or a “worrier”, as well as parents who described their parenting style as overprotective, the uncertainty related to child safety appeared particularly difficult to overcome.

#### Peers and siblings

Also situated closely within the child’s environment are siblings and peers [15]. The benefits of playing with siblings and peers have been seen for various aspects of development, including social competence [52]. Interestingly, evidence also relates the presence of peers and siblings to *increased* engagement in injury-risk behaviours. Several studies have documented that the presence of siblings and peers significantly increases children’s risk-taking decisions that carry a greater chance of injury [53, 54]. This could be partially explained by the influence of positive sibling relationships or peer group social norms [55, 56], with some evidence showing that children reported their risk-taking was influenced by peer group expectations and a desire to ‘fit in’ [57]. Given this evidence, it may be surprising that parents in the current study reported the presence of peers or older siblings as facilitators of adventurous play. This highlights that for some parents, the mere presence of a peer or sibling may operate to increase perceptions of safety due to there being another person to help the child if required. This may override concerns about the possibility of increased risk-taking. A sense of safety in numbers has been described by both parents [58] and young people [59] when considering independent and outdoor play and therefore may apply similarly to adventurous play.

#### The built environment

The built environment represents a more distal influence from the child in the context of Bronfenbrenner’s model [15]. Nonetheless, it has been shown to affect children’s play opportunities as well as parents’ perceptions of safety in relation to children’s play [60, 61]. Urbanisation is one aspect of the built environment that has relevance here. In Britain urbanisation is high, with 81.5% of people living in England and Wales in 2011 estimated to live in urban areas [62]. Increased traffic, fewer green spaces and reduced neighbourhood communal areas due to urbanisation [63] can impact perceptions of neighbourhood connectedness [64]. There can also be consequences for children’s access to nature and limitations to their free play opportunities, by reducing the space to play and making safe access to play spaces more challenging [65, 66]. It therefore also influences parents’ perceptions of safety in their local area, which adds further limitations to children’s play opportunities [60].

This influence was evident in some parent’s responses in the current study in relation to both the physical and social environment. An absence of community, poor accessibility to play spaces as well as the challenges of busy roads and increased car usage were sometimes cited as barriers. Our findings highlight the importance of responsibility being placed not only on parents, but also on higher-level agents, including urban and transport planners, to improve children’s adventurous play opportunities [60, 61]. The design and planning of spaces, including play spaces and neighbourhoods, has the potential to ‘nudge’ parents towards encouraging more adventurous play opportunities [67]; when parents perceive the environment as safer, it is likely they will grant their children more freedoms [60].

#### Cultural factors

Cultural attitudes and ideologies are distally related to children and their parents [15] and it is unsurprising that parents’ responses in this study rarely related to cultural influences. Certain westernised societies, such as Britain, are recognised as risk-averse cultures, with a stance of surplus safety in relation to child risk-taking [9, 61]. Media agents can reinforce cultural ideologies and attitudes by reporting on and therefore highlighting the rare consequences of taking risks, such as severe physical injury or stories involving child abduction. Such reporting influences perceptions of risk and the safety of society [61, 68].

In this study, we found a dominating positive attitude towards adventurous play that perhaps may be unexpected given the culture of risk-averseness. It could be that the influence of cultural attitudes and ideologies on parents affects behaviour but positive beliefs about adventurous play remain. In this study, themes related to the safety of society, the risk of “stranger danger” and judgement from others as barriers could to some extent be implicitly related to the overall attitudes and ideologies of the culture. There were also some explicit mentions of the media as a barrier, which causes parents to question whether they should allow their children to take risks in their play or not.

### Implications for policy and intervention

**Fig. 2:**
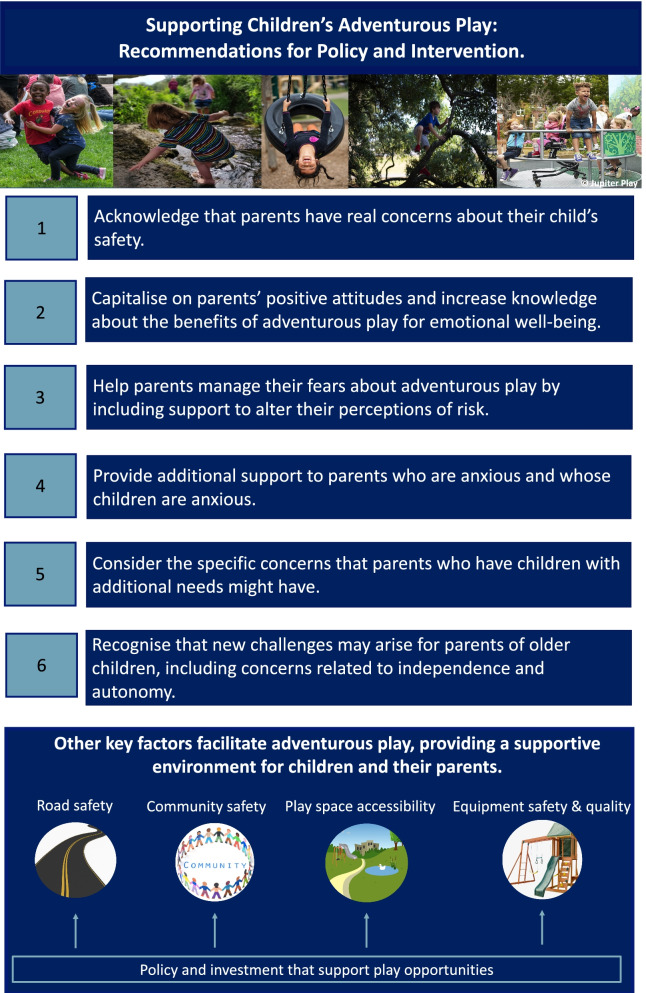
Recommendations for policy and interventions

In view of this study’s findings and the documented benefits of adventurous play for children’s physical and mental health [5, 10], several recommendations for policy and intervention are proposed. These are shown in Fig. 2. Given that most parents experienced barriers in relation to their child’s safety, parents may benefit from support to facilitate adventurous play, with some needing more support than others. We recommend that this support is offered via public health interventions and campaigns aimed at parents.

Types of support that may be beneficial for parents includes risk-reframing, which aims to develop an increased tolerance of risk in children’s play and address the tension between positive attitudes towards adventurous play and the competing barriers. Some preliminary successes have been seen in risk-reframing interventions [69, 70]. Further, working with parents to manage their cognitions around uncertainty may be valuable, perhaps by encouraging exposure to uncertainty in their children’s play. Adults working in a school have previously reported that when ‘stepping back’ instead of ‘stepping in’ during children’s risky play, they were able to observe children’s competencies in self-management of risks [71].

### Strengths and limitations

Using open-ended questions allowed us to analyse data from a large, diverse sample of parents, which is a key strength. The limitation of this approach is that there was a lack of depth to responses and it was not possible for us to establish a shared understanding with the participants. Employing alternative qualitative methods, for example one-to-one semi-structured interviews would likely have generated richer data. However, it would have restricted the sample to fewer participants, and given the under-representation of specific population sub-groups in research, we would have failed to capture the same breadth and diversity of perspectives [72].

Another important strength is the use of Framework Analysis, which yielded findings that captured the breadth of factors parents perceived as barriers and facilitators to adventurous play. Because of this detail, we chose to focus the discussion on barriers and facilitators that have particular relevance for intervention but this means that some, such as practical considerations (time, weather, younger siblings), were not discussed in-depth. Nevertheless, they are important for some parents, and we would recommend they are not overlooked in the development of policy and intervention.

A final strength is that the identified framework has utility for future research, providing a potential structure for quantitative research to explore the barriers and facilitators most commonly perceived by parents and therefore identify the most important targets for intervention. Applying the framework structure quantitatively to the wider 1919 sample of parents in the BCPS could be considered.

## Conclusions

Given that adventurous play has been associated with children’s development, physical and mental health, the current study aimed to identify the barriers and facilitators of adventurous play perceived by parents of primary school-aged children in Britain. The findings highlight the breadth of factors operating to influence parents’ encouragement or restriction of adventurous play. Interventions and campaigns that respond to parents’ concerns, capitalise on existing positive attitudes and support parents with tolerating risk and uncertainty may be important to target, with a view to ultimately improve children’s adventurous play opportunities, as well as their health.

## Supplementary Information

**Additional file 1.** Further details on how parents were sampled.

## Data Availability

The data obtained and analysed in the current study are available via the following link: 10.5255/UKDA-SN-8793-1.

## Abbreviations

BCPS: British Children’s Play Survey
