# Supplementary material for: Parent perceived barriers and facilitators of children’s adventurous play in Britain: a framework analysis
Source: BMC Public Health. 2022 Apr 1;22:636. doi: 10.1186/s12889-022-13019-w (PMC8976306; doi:10.1186/s12889-022-13019-w)
Supplement: Supplementary file 1 — Additional file 1. Further details on how parents were sampled. [file 12889_2022_13019_MOESM1_ESM.docx]

**Supplementary Information**

**Additional file 1 includes:**

- Written information describing how parents and caregivers were sampled
- Written information describing how the key characteristics of parents and caregivers were categorised
- A table showing the clusters of parents and caregivers sampled

**Written information describing how parents and caregivers were sampled:**

To select a subsample, parents were grouped into clusters based on the key characteristics. For example, one cluster was mothers, of younger (aged 5-7) boys, with lower education and who were White British. Having identified clusters of the five characteristics, we selected the first four parents in the survey datafile for each cluster to ensure good representation of each, although some clusters contained fewer than four parents. Once sampling was completed, we checked the identified sample (n=368) for diversity in terms of other demographic characteristics (e.g. relationship to child, parent marital status). Diversity in the sample was achieved except that no foster parents or widowed parents had been selected. In the whole sample there were five foster parents and four widowed parents. We chose to include all of these in our sample as they were diverse in terms of the key characteristics.

**Written information describing how the key characteristics of parents and caregivers were categorised:**

In the survey, parents were asked to provide a range of socio-demographic and geographic information. To categorise child age group*, (Younger: 5-7-years-old; Older: 8-11-years-old)*, we used the approximate age boundaries of infant school and junior school (1).

To categorise parent ethnicity, responses were condensed into seven categories, as per the Office for National Statistics Annual Population Survey (2). The seventh category was included to represent the substantial number of parents who chose not to provide data on their ethnicity.  Ethnicity was characterised as: *White British; Any other White ethnicity; Mixed or Multiple ethnic groups; Asian or Asian British; Black or Black British; Other ethnic group; No ethnicity information*.

For education level, responses were condensed into *Lower; Medium; Higher* using the categorisation system used by YouGov, the UK public opinion research company from which the British Children’s Play Survey recruited parents (for more information, see Dodd & Colleagues (3)).

References:

1. Department for Education. Education and Training Statistics for the United Kingdom: 2017. 2017. <https://assets.publishing.service.gov.uk/government/uploads/system/uploads/attachment_data/file/657822/SFR64_2017_Additional_Text.pdf>

2. Office for National Statistics. Population Estimates by Ethnic Group. 2017.

<https://www.ons.gov.uk/peoplepopulationandcommunity/populationandmigration/populationestimates/methodologies/populationestimatesbyethnicgroup>

3. Dodd HF, FitzGibbon L, Watson BE, Nesbit RJ. Children’s Play and Independent Mobility in 2020: Results from the British Children’s Play Survey. International Journal of Environmental Research and Public Health. 2021;18(8):4334. <https://doi.org/https://doi.org/10.3390/ijerph18084334>

**A table showing the clusters of parents and caregivers sampled:**

| **Child age (Younger/Older)** | **Child sex (Male/Female)** | **Parent sex (Mother/ Father)** | **Ethnicity** | **Education level (Lower/Medium/ Higher)** |
| --- | --- | --- | --- | --- |
| Younger | Male | Mother | White British | Lower |
| Older | Male | Mother | White British | Lower |
| Younger | Female | Mother | White British | Lower |
| Older | Female | Mother | White British | Lower |
| Younger | Male | Father | White British | Lower |
| Older | Male | Father | White British | Lower |
| Younger | Female | Father | White British | Lower |
| Older | Female | Father | White British | Lower |
| Younger | Male | Mother | Other White | Lower |
| Older | Male | Mother | Other White | Lower |
| Younger | Female | Mother | Other White | Lower |
| Older | Female | Mother | Other White | Lower |
| Younger | Male | Father | Other White | Lower |
| Older | Male | Father | Other White | Lower |
| Younger | Female | Father | Other White | Lower |
| Older | Female | Father | Other White | Lower |
| Younger | Male | Mother | Mixed | Lower |
| Older | Male | Mother | Mixed | Lower |
| Younger | Female | Mother | Mixed | Lower |
| Older | Female | Mother | Mixed | Lower |
| Younger | Male | Father | Mixed | Lower |
| Older | Male | Father | Mixed | Lower |
| Younger | Female | Father | Mixed | Lower |
| Older | Female | Father | Mixed | Lower |
| Younger | Male | Mother | Asian | Lower |
| Older | Male | Mother | Asian | Lower |
| Younger | Female | Mother | Asian | Lower |
| Older | Female | Mother | Asian | Lower |
| Younger | Male | Father | Asian | Lower |
| Older | Male | Father | Asian | Lower |
| Younger | Female | Father | Asian | Lower |
| Older | Female | Father | Asian | Lower |
| Younger | Male | Mother | Black | Lower |
| Older | Male | Mother | Black | Lower |
| Younger | Female | Mother | Black | Lower |
| Older | Female | Mother | Black | Lower |
| Younger | Male | Father | Black | Lower |
| Older | Male | Father | Black | Lower |
| Younger | Female | Father | Black | Lower |
| Older | Female | Father | Black | Lower |
| Younger | Male | Mother | Other | Lower |
| Older | Male | Mother | Other | Lower |
| Younger | Female | Mother | Other | Lower |
| Older | Female | Mother | Other | Lower |
| Younger | Male | Father | Other | Lower |
| Older | Male | Father | Other | Lower |
| Younger | Female | Father | Other | Lower |
| Older | Female | Father | Other | Lower |
| Younger | Male | Mother | White British | Medium |
| Older | Male | Mother | White British | Medium |
| Younger | Female | Mother | White British | Medium |
| Older | Female | Mother | White British | Medium |
| Younger | Male | Father | White British | Medium |
| Older | Male | Father | White British | Medium |
| Younger | Female | Father | White British | Medium |
| Older | Female | Father | White British | Medium |
| Younger | Male | Mother | Other White | Medium |
| Older | Male | Mother | Other White | Medium |
| Younger | Female | Mother | Other White | Medium |
| Older | Female | Mother | Other White | Medium |
| Younger | Male | Father | Other White | Medium |
| Older | Male | Father | Other White | Medium |
| Younger | Female | Father | Other White | Medium |
| Older | Female | Father | Other White | Medium |
| Younger | Male | Mother | Mixed | Medium |
| Older | Male | Mother | Mixed | Medium |
| Younger | Female | Mother | Mixed | Medium |
| Older | Female | Mother | Mixed | Medium |
| Younger | Male | Father | Mixed | Medium |
| Older | Male | Father | Mixed | Medium |
| Younger | Female | Father | Mixed | Medium |
| Older | Female | Father | Mixed | Medium |
| Younger | Male | Mother | Asian | Medium |
| Older | Male | Mother | Asian | Medium |
| Younger | Female | Mother | Asian | Medium |
| Older | Female | Mother | Asian | Medium |
| Younger | Male | Father | Asian | Medium |
| Older | Male | Father | Asian | Medium |
| Younger | Female | Father | Asian | Medium |
| Older | Female | Father | Asian | Medium |
| Younger | Male | Mother | Black | Medium |
| Older | Male | Mother | Black | Medium |
| Younger | Female | Mother | Black | Medium |
| Older | Female | Mother | Black | Medium |
| Younger | Male | Father | Black | Medium |
| Older | Male | Father | Black | Medium |
| Younger | Female | Father | Black | Medium |
| Older | Female | Father | Black | Medium |
| Younger | Male | Mother | Other | Medium |
| Older | Male | Mother | Other | Medium |
| Younger | Female | Mother | Other | Medium |
| Older | Female | Mother | Other | Medium |
| Younger | Male | Father | Other | Medium |
| Older | Male | Father | Other | Medium |
| Younger | Female | Father | Other | Medium |
| Older | Female | Father | Other | Medium |
| Younger | Male | Mother | White British | Higher |
| Older | Male | Mother | White British | Higher |
| Younger | Female | Mother | White British | Higher |
| Older | Female | Mother | White British | Higher |
| Younger | Male | Father | White British | Higher |
| Older | Male | Father | White British | Higher |
| Younger | Female | Father | White British | Higher |
| Older | Female | Father | White British | Higher |
| Younger | Male | Mother | Other White | Higher |
| Older | Male | Mother | Other White | Higher |
| Younger | Female | Mother | Other White | Higher |
| Older | Female | Mother | Other White | Higher |
| Younger | Male | Father | Other White | Higher |
| Older | Male | Father | Other White | Higher |
| Younger | Female | Father | Other White | Higher |
| Older | Female | Father | Other White | Higher |
| Younger | Male | Mother | Mixed | Higher |
| Older | Male | Mother | Mixed | Higher |
| Younger | Female | Mother | Mixed | Higher |
| Older | Female | Mother | Mixed | Higher |
| Younger | Male | Father | Mixed | Higher |
| Older | Male | Father | Mixed | Higher |
| Younger | Female | Father | Mixed | Higher |
| Older | Female | Father | Mixed | Higher |
| Younger | Male | Mother | Asian | Higher |
| Older | Male | Mother | Asian | Higher |
| Younger | Female | Mother | Asian | Higher |
| Older | Female | Mother | Asian | Higher |
| Younger | Male | Father | Asian | Higher |
| Older | Male | Father | Asian | Higher |
| Younger | Female | Father | Asian | Higher |
| Older | Female | Father | Asian | Higher |
| Younger | Male | Mother | Black | Higher |
| Older | Male | Mother | Black | Higher |
| Younger | Female | Mother | Black | Higher |
| Older | Female | Mother | Black | Higher |
| Younger | Male | Father | Black | Higher |
| Older | Male | Father | Black | Higher |
| Younger | Female | Father | Black | Higher |
| Older | Female | Father | Black | Higher |
| Younger | Male | Mother | Other | Higher |
| Older | Male | Mother | Other | Higher |
| Younger | Female | Mother | Other | Higher |
| Older | Female | Mother | Other | Higher |
| Younger | Male | Father | Other | Higher |
| Older | Male | Father | Other | Higher |
| Younger | Female | Father | Other | Higher |
| Older | Female | Father | Other | Higher |
| Younger | Male | Mother | No info | Lower |
| Older | Male | Mother | No info | Lower |
| Younger | Female | Mother | No info | Lower |
| Older | Female | Mother | No info | Lower |
| Younger | Male | Father | No info | Lower |
| Older | Male | Father | No info | Lower |
| Younger | Female | Father | No info | Lower |
| Older | Female | Father | No info | Lower |
| Younger | Male | Mother | No info | Medium |
| Older | Male | Mother | No info | Medium |
| Younger | Female | Mother | No info | Medium |
| Older | Female | Mother | No info | Medium |
| Younger | Male | Father | No info | Medium |
| Older | Male | Father | No info | Medium |
| Younger | Female | Father | No info | Medium |
| Older | Female | Father | No info | Medium |
| Younger | Male | Mother | No info | Higher |
| Older | Male | Mother | No info | Higher |
| Younger | Female | Mother | No info | Higher |
| Older | Female | Mother | No info | Higher |
| Younger | Male | Father | No info | Higher |
| Older | Male | Father | No info | Higher |
| Younger | Female | Father | No info | Higher |
| Older | Female | Father | No info | Higher |
